# Soil Bacterial Community Shifts Are Driven by Soil Nutrient Availability along a Teak Plantation Chronosequence in Tropical Forests in China

**DOI:** 10.3390/biology10121329

**Published:** 2021-12-15

**Authors:** Zhi Yu, Kunnan Liang, Guihua Huang, Xianbang Wang, Mingping Lin, Yinglong Chen, Zaizhi Zhou

**Affiliations:** 1 Key Laboratory of State Forestry Administration on Tropical Forestry Research, Research Institute of Tropical Forestry, Chinese Academy of Forestry, Guangzhou 510520, China; yuzhi6000@163.com (Z.Y.); wangxb@caf.ac.cn (X.W.); jfllinmingping@126.com (M.L.); zzzhoucn@21cn.com (Z.Z.); 2 Institute of Agriculture, School of Agriculture and Environment, The University of Western Australia, Perth, WA 6009, Australia; yinglong.chen@uwa.edu.au

**Keywords:** *Tectona grandis*, co-occurrence patterns, soil bacterial structure, chronosequence, rhizosphere soil, bulk soil, bacterial community, bacterial diversity, ecosystem services, succession

## Abstract

**Simple Summary:**

Tropical forests play an important role in the global carbon cycle, especially in the context of global climate change. Soil microorganisms are essential to the functions, services, and productivity of terrestrial ecosystems as a link to maintain the connections and interactions between the aboveground and belowground ecosystems. The interactions between plants and the soil microbiome are crucial for plant growth, health, and resistance to stressors. However, information on the response of soil microbial communities to a chronosequence of woody plants is lacking, especially in tropical forests. This study compares the soil properties, diversity, composition, and co-occurrence patterns of bacterial communities in the rhizosphere and bulk soils along a teak plantation chronosequence. The results show that the composition and co-occurrence patterns of the bacterial communities are statistically different among the plantations, while stand age has no significant impact on soil bacterial alpha diversity. The results further show that soil nutrients play a key role in shaping the soil bacterial community. The study also provides information about the dynamics and characteristics of these soil bacterial communities and adds valuable information that may underpin new strategies for the management of teak plantations.

**Abstract:**

Soil bacterial communities play crucial roles in ecosystem functions and biogeochemical cycles of fundamental elements and are sensitive to environmental changes. However, the response of soil bacterial communities to chronosequence in tropical ecosystems is still poorly understood. This study characterized the structures and co-occurrence patterns of soil bacterial communities in rhizosphere and bulk soils along a chronosequence of teak plantations and adjacent native grassland as control. Stand ages significantly shifted the structure of soil bacterial communities but had no significant impact on bacterial community diversity. Bacterial community diversity in bulk soils was significantly higher than that in rhizosphere soils. The number of nodes and edges in the bacterial co-occurrence network first increased and then decreased with the chronosequence. The number of strongly positive correlations per network was much higher than negative correlations. Available potassium, total potassium, and available phosphorus were significant factors influencing the structure of the bacterial community in bulk soils. In contrast, urease, total potassium, pH, and total phosphorus were significant factors affecting the structure of the bacterial community in the rhizosphere soils. These results indicate that available nutrients in the soil are the main drivers regulating soil bacterial community variation along a teak plantation chronosequence.

## 1. Introduction

As engines of Earth systems, tropical forests play a critical role in the global carbon and water cycle, especially in the context of global climate change [1]. The biodiversity and ecosystem functions of tropical forests are important for human survival and development [2]. The connections and interactions between aboveground and belowground are crucial to the structure and function of ecosystems and are one of the important driving forces for ecosystem services, such as plant productivity and storage of nutrients and water [3]. Soil microbes are the link that maintains the connections and interactions between aboveground and belowground terrestrial ecosystems [4]. Forest succession, as a normal phenomenon, may influence the nature of ecosystems via many components, such as changes in the composition and diversity of vegetation, microclimate under the forest, decomposition and accumulation of litter, and soil characteristics [5]. These changes may drive shifts in microbial communities [6,7]. In turn, microbes play a vital part in shaping the aboveground biodiversity and functions and services of terrestrial ecosystems by driving crucial biogeochemical processes and mediating the nutrient cycle [8,9,10]. For instance, nitrogen-fixing bacteria, mycorrhizal fungi, and rhizosphere growth-promoting bacteria can directly enhance the ability of plants to obtain nutrients [11]. Moreover, soil microorganisms can affect the process of soil nutrient cycling, improve the availability of soil nutrients, reduce nutrient loss, and indirectly influence the number of plant nutrients obtained, especially in the rhizosphere, which is the area of soil around the plant roots directly affected by rhizodeposition of exudates, residues, and sloughed cells [12,13,14]. Rhizosphere microorganisms are associated with plant growth, development, health, and stress tolerance [15]. Therefore, the rhizosphere microbiome resembles the second genome of plants [16], and it functions similarly to the gut microbes in the human body. Hence, exploring the soil microbial community dynamics, characteristics, and potential driving force in forest succession may provide new insights into the sustainable management of forests.

One of the central questions in soil microbial ecology is how soil microbial communities respond to ecological succession [10] and what are the driving forces. Mounting evidence in the literature has shown that the bacterial community structure is shaped by multiple abiotic and biotic factors, such as pH, nutrient availability, soil moisture, plant diversity, and underground vegetation characteristics [9]. Prior studies reported that soil properties, especially pH, exerted a strong effect on the composition and structure of bacterial communities [17]. Thus, factors that can influence soil pH may indirectly result in variations in bacterial community composition and structure. Zhao et al. [18] recently reported that bacterial communities generally changed from Acidobacteria-dominant to Proteobacteria-dominant during the 30 years of succession on the Loess Plateau, correlating with the levels of soil nutrients. Liu et al. [19] also revealed that the bacterial community structures in the rhizosphere soil of *Pinus tabulaeformis* were significantly associated with total phosphorous, total nitrogen, and available phosphorous concentrations. The shift in the composition and diversity of underground vegetation during forest succession can simultaneously contribute to variations in bacterial community composition and structure [20]. For example, leaf litter quality and quantity determined by tree species can significantly alter soil chemical properties [21], enabling shifts in bacterial community composition. It is generally believed that microbial community assembly is scale-dependent [22]. On a local scale, abiotic factors (litter, soil nutrients, soil pH, and temperature) and biotic factors (underground vegetation characteristics) jointly explain the variation in soil bacterial community composition [5,6,9]. A study conducted on the Loess Plateau found that the contributions of soil and plants can jointly explain 76.95% of soil bacterial diversity and 90.64% of bacterial composition during the second succession [23].

In the natural environment, microbes usually live within complex ecological networks that interact with each other [24,25] and include positive interactions, such as competition and predation, and negative interactions, such as commensalism and mutualism. [26]. Most analytical methods of soil microbial communities have been used to explain microbial diversity, composition, and the relationship between their changes and environmental factors [27], however, the relationships among the soil microbial species are still not clear. Recently, co-occurrence network analysis has been widely applied to characterize ecological linkages among microbes in complex ecosystems, including rivers [28], caves [29], soil [8,26], phyllosphere [25,30], the gut microbiome [31], and ocean ecosystems [32]. Co-occurrence network analysis can shed light on the mechanism of structure and assembly of complex microbial communities [33].

Teak (*Tectona grandis*), one of the most expensive hardwoods in the world, is naturally distributed in India, Myanmar, Laos, and Thailand [34]. Teak was introduced into areas of tropical and southern subtropical China due to its increased economic and social value, since the 1990s [35]. Teak generally grows well in tropical and southern subtropical China, but often suffers from biotic and abiotic stressors, such as drought, low temperatures, and diseases. Considering the close relationship between soil bacterial communities and plant productivity, exploring the diversity, composition, and co-occurrence patterns of soil bacterial communities with stand ages may provide new strategies for the cultivation of teak.

The objectives of this study were to illustrate the regulation of, and the variations in the soil bacterial community along a teak plantation chronosequence, and specifically the effects of stand age using space-for-time substitution. We hypothesized that (1) teak plantations can significantly influence soil bacterial diversity, community composition, and co-occurrence pattern, and (2) soil properties may be a direct environmental factor influencing the soil bacterial community.

## 2. Materials and Methods

### 2.1. Study Region

The study was conducted at Mt. Jianfengling (18°20′–18°57′ N, 108°41′–109°12′ E) in Hainan, China. The site is characterized by a tropical monsoon climate, with an annual average temperature of 24.5 °C. The annual extreme maximum and minimum temperatures are 38.1 °C and 2.5 °C, respectively. The whole year is divided into two seasons: the rainy season from May to October, and the dry season from November to April of the following year. The average annual rainfall is 2449 mm, and the relative humidity varies between 80% and 88%.

### 2.2. Experimental Design and Soil Sample Collection

The study was conducted in December 2020. Based on relevant afforestation information from the Research Institute of Tropical Forestry, Chinese Academy of Forestry, four age groups, 22 years (22-y), 35 years (35-y), 45 years (45-y), and 55 years (55-y) of the teak plantation, not subjected to management interference, were selected as the experimental units. In addition, a nearby native grassland, with no history of teak plantation, was sampled as a control (CK). *Heteropogon contortus*, a tropical grass species, dominated the grassland. Since the distribution of *H. contortus* was dense, it was difficult to distinguish the bulk soils in the grassland and we, therefore, only used rhizosphere soils in the nearby native grassland as a control. The environmental characteristics of the experimental sites were similar, and the stand characteristics selected were measured as shown in Table 1.

Three standard sites with dimensions of 20 × 20 m^2^ were set up for each age group, and five dominant teak trees in each standard site were selected as experimental objects and marked with red paint. The distance between these standard sites was less than 100 m, and the stand distance of different ages was less than 5 km. The top 5 cm of soil was removed from each selected site to obtain exposed roots, and each root was followed to its origin. The rhizosphere soil was defined as soil tightly adhering to the root surface, which was collected using a sterile shovel and a paintbrush. Bulk soil was collected from the unvegetated soil at a depth of 0–10 cm and was not directly attached to the root systems [36]. All rhizosphere or bulk soil samples from each site were mixed to form a composite sample. Each of the rhizosphere and bulk soil samples was separated into two parts: one part of the soil was passed through a 2 mm sieve after being air-dried in the laboratory and used for soil enzyme activity and chemical property analysis. The other part was kept in a freezer at −80 °C for DNA extraction. A total of 15 rhizosphere soil samples (12 samples from four teak stands and three CK samples) and 12 bulk soil samples (no bulk CK samples were collected) were obtained.

### 2.3. Soil DNA Isolation and PCR Amplification

DNA extraction from 0.3 g frozen rhizosphere and bulk soil samples was performed using the E.Z.N.A.^®^ soil DNA Kit (Omega Bio-Tek, Norcross, GA, USA) according to the manufacturer’s instructions. After extraction, the DNA concentration and purity were determined using a NanoDrop 2000 UV-Vis spectrophotometer (Thermo Scientific, Wilmington, NC, USA) and 1% agarose gel. The 16s rRNA V3-V4 region of the bacteria was used as the target sequence, and PCR amplification was carried out using the primer pairs 338F (5′-ACTCCTACGGGAGGCAGCAG-3′) and 806R (5′-GGACTACHVGGGTWTCTAAT-3′) [5] using an ABI GeneAmp^®^ 9700 PCR thermocycler (ABI, Foster City, CA, USA). The amplification reaction mixture contained 4 μL of the 5 × FastPfu Buffer, 2 μL of 2 mM dNTPs, 0.8 μL of the forward primer (5 μM), 0.8 μL of the reverse primer (5 μM), 0.4 μL of the FastPfu Polymerase, 0.2 μL BSA, 10 ng template DNA, and ddH_2_O added to a final reaction mixture volume of 20 μL. PCR reactions were performed in triplicate. PCR amplification conditions were an initial denaturation at 95 °C for 3 min, followed by 27 cycles of denaturation at 95 °C for 30 s, annealing at 55 °C for 30 s, and extension at 72 °C for 45 s, with a final extension at 72 °C for 10 min.

### 2.4. Illumina Miseq Sequencing

The PCR amplification product was extracted using a 2% agarose gel and purified using an AxyPrep DNA Gel Extraction Kit (Axygen Biosciences, Union City, CA, USA). Purified amplicons were pooled in equimolar amounts and paired-end sequenced on an Illumina Miseq PE 300 platform (Illumina, San Diego, CA, USA) according to the standard protocols of Majorio Bio-Pharm Technology Co., Ltd. (Shanghai, China). The raw reads for all samples were sent to the NCBI Sequence Read Archive (SRA) database (accession number: PRJNA752823).

### 2.5. Processing of Sequencing Data

The raw reads were demultiplexed, quality-filtered using fastq version 0.20.0 [37], and merged using FLASH version 1.2.7 [38]. Operational taxonomic units (OTUs) were clustered using UPARAE version 7.1 (http://drive5.com/uparse/, accessed on 8 August 2021) based on 97% identity, and chimeric sequences were identified and removed. The taxonomy of each OTU representative sequence was assigned using the Ribosomal Database Project (RDP) classifier (http://rdp.cme.msu.edu/, accessed on 8 August 2021) against the 16S rRNA Sliva (v132) database using a confidence threshold of 0.9.

### 2.6. Soil Physicochemical Parameters and Enzyme Activity Measurements

Soil parameters were determined according to standard soil test methods, as reported by the agricultural protocols for China [39]. Soil pH was measured using a glass electrode pH meter (soil to water ratio was 1:2.5). The soil organic carbon (SOC) content was measured using the high-temperature external heat dichromate oxidation capacity method. Total nitrogen (TN) was assessed using the Kjeldahl digestion method. Total phosphorus (TP) was measured using the Mo-Sb anti-spectrophotometric method. Total potassium (TK) was determined using the atomic absorption method (Z-2300, Tokyo, Japan). Available phosphorus (AP) was extracted using HCl-NH_4_F solution and determined using the molybdenum-antimony resistance colorimetric method. Available potassium (AK) was extracted with ammonium acetate and measured using flame absorption spectroscopy (Z-2300, Tokyo, Japan). The soil ammonium-N (${\mathrm{NH}}_{4}^{+}$-H) and nitrate-N (${\mathrm{NO}}_{3}^{-}$-H) were extracted with KCl solution and measured using indophenol blue colorimetry and two-wavelength ultraviolet spectrometry [40]. The activities of soil urease, acid phosphatase, and catalase were determined as reported by Zhen et al. [41].

### 2.7. Statistical Analysis

Tukey’s honestly significant difference (HSD) test and one-way analysis of variance (ANOVA) were used to determine the differences in soil chemical properties, diversity indices, and the relative abundance of major bacterial phyla. The relative abundance of bacterial phyla were normalized with log _10_ (X + 1) conversion before assessment. The alpha diversity indices, including Sobs (number of observed OTUs), Chao 1, Shannon, and Simpson, were analyzed using Mothur (version v.1.30.2, http://www.mothur.org/wiki/Schloss_SOP#Alpha_diversity, accessed on 8 August 2021). The relationships among the bacterial communities in the teak plantations of different stand ages were analyzed by non-metric multidimensional scaling (NMDS) using the Vegan package in R studio. Analysis of similarities (ANOSIM) and permutation multivariate analysis of variance (PERMANOVA) were conducted to test the statistically significant differences among bacterial communities with Bray–Curtis distances and 999 permutations. Redundancy analysis (RDA) and Mantel test with 999 permutations were used to identify the effects of soil properties on the bacterial communities using vegan and ade4 packages in R studio. Spearman’s correlation analysis was used to estimate the relationship between bacterial alpha diversity and soil properties using R studio. To reveal bacterial composition differences between rhizosphere and bulk soil along a teak plantation chronosequence, we used the following formula for bacterial dominant phyla:

Differences in dominant bacterial phyla

Difference value = ${\mathrm{Rhi}}_{\mathrm{phyla}}$ − ${\mathrm{Bulk}}_{\mathrm{phyla}}$ (Rhi_phyla_: abundance of the dominant phyla in rhizosphere soils; Bulk_phyla_: abundance of the dominant phyla in bulk soils)

Network analysis was applied to determine the co-occurrence patterns of bacterial communities among the different stand ages of the teak plantations. The analysis was performed using all correlations of the 100 most abundant OTUs in the soil bacterial community [27,42]. At every growth stage, the samples originated from the rhizosphere and bulk soil. Co-occurring networks based on Pearson’s correlation analysis in this study were performed using the psych package in R studio [25]. The co-occurrence patterns of soil bacterial communities were studied based on strong correlations (correlation coefficient R > 0.6) and significant correlations (*p <* 0.01). Visualization of networks and calculation of network topological parameters were performed using the interactive platform Gephi [29].

## 3. Results

### 3.1. Soil Physicochemical Properties

At the 35-y sites, pH, SOC, TN, TP, AK, catalase, acid phosphatase, and urease were significantly (*p <* 0.05) higher than at other sampling sites in the rhizosphere soils. TK at the 22-y sites was higher than at other sampling sites in both the rhizosphere and bulk soils. The ${\mathrm{NO}}_{3}^{-}$-H, ${\mathrm{NH}}_{4}^{+}$-H, and AP were not significantly different among the five stand ages in the rhizosphere. The SOC, AK, and catalase in the bulk soils all increased first and then decreased with forest succession, peaking at the 35-y sites, while the ${\mathrm{NO}}_{3}^{-}$-H showed an increasing trend with stand age, peaking at 55-y sites (Table S1).

### 3.2. Changes in Soil Bacterial Alpha Diversity

**Figure 1:**
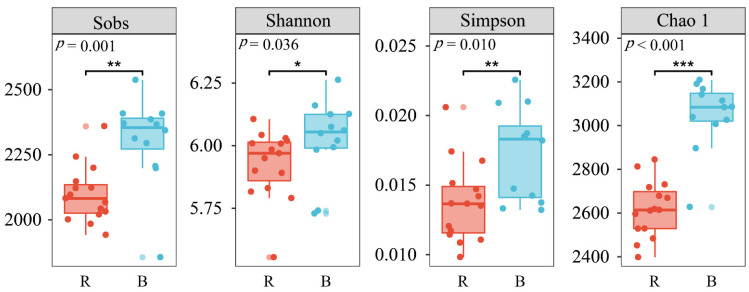
Alpha diversity indices of soil bacterial communities were compared between bulk (*n* = 12) and rhizosphere (*n* = 15) soils based on Kruskal-Wallis test. R: rhizosphere soil; B: bulk soil. * = *p* < 0.05; ** = *p* < 0.01; *** = *p* < 0.001.

A total of 406,273 and 483,838 high-quality bacterial sequences were identified from the 12 bulk soil samples and 15 rhizosphere soil samples, and the bacterial sequences were clustered into 4820 and 3400 OTUs, respectively. The Sobs, Chao 1, Shannon, and Simpson indices among the different stand groups were not significantly (*p* > 0.05) different in the bulk and rhizosphere soils. Coverage was above 97% for each sample, suggesting that soil bacterial communities were well sampled owing to the depth of Illumina sequencing (Table 2). However, the Sobs, Shannon, Simpson, and Chao 1 indices were significantly higher in the bulk soils than in the rhizosphere soils (Figure 1).

### 3.3. Soil Bacterial Community Composition and Structure with Stand Development

**Figure 2:**
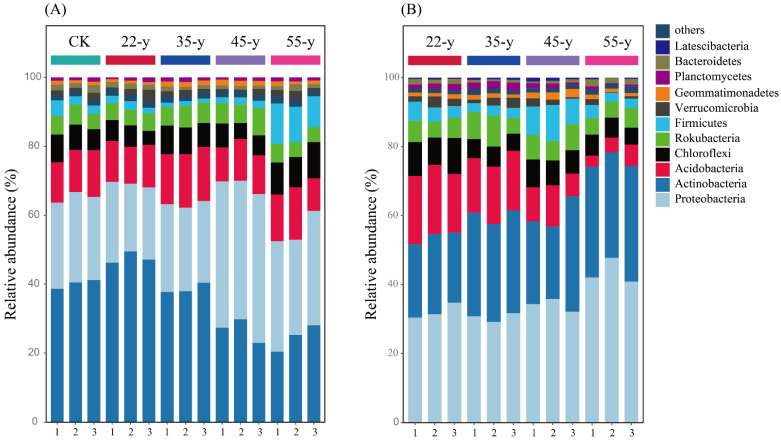
The relative abundance of bacterial phyla in bulk (**B**) and rhizosphere (**A**) soils of teak plantations with different stand ages.

**Figure 3:**
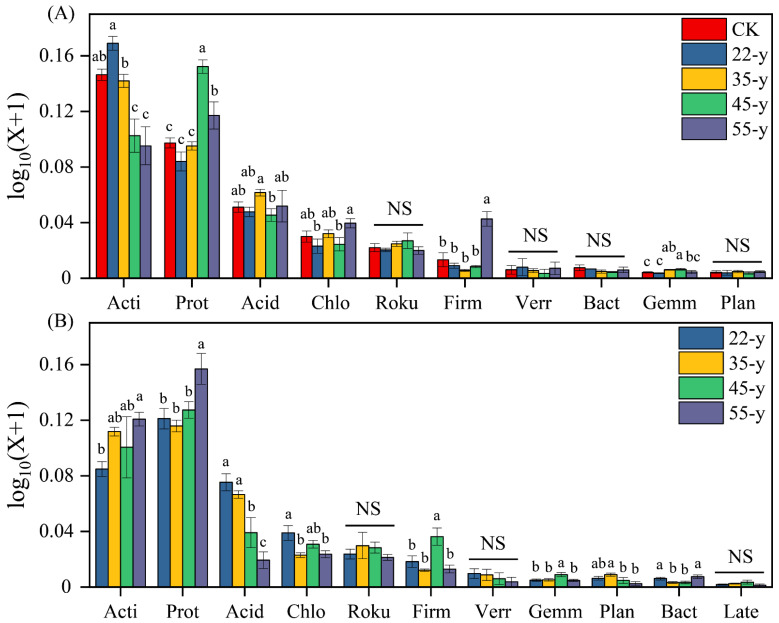
Relative abundance of soil bacterial communities at the phylum level between rhizosphere (**A**) and bulk (**B**) soils along a gradient of teak plantations. Letters indicate significant difference at 0.05 levels among different stand ages. Ns = not significance (*p* > 0.05). Acti: Actinobacterial; Prot: Proteobacteria; Aci-d: Acidobacteria; Chlo: chloroflexi; Roku: Rokubacteria; Firm: Firmicutes; Verr: Verrucomicrobia; Bact: Bacteroidetes; Gemm: Gemmatimonadetes; Plan: Planctomycetes.

The dominant bacterial phyla were Actinobacteria (35.53%), Proteobacteria (28.79%), Acidobacteria (12.63%), and Chloroflexi (7.13%), whereas Rokubacteria, Firmicutes, Verrucomicrobia, and other bacterial species occupied only a minor fraction of the bacterial community composition in the rhizosphere soils (Figure 2A). The most dominant bacterial phyla in bulk soil of the teak plantations were Proteobacteria (35.11%), Actinobacteria (27.31%), Acidobacteria (12.39%), and Chloroflexi (6.94%). Other bacterial phyla, such as Planctomycetes, Verrucomicrobia, and Geommatimonadetes accounted for only a minor fraction of the bulk soil community composition (Figure 2B). Regarding the differences in bacterial community composition between the rhizosphere and bulk soils (Figure S1), Actinobacteria in rhizosphere soils were more abundant except in the 55-y sites, and the difference showed an initial decrease and then an increasing trend as stand age progressed. Proteobacteria were more abundant in bulk soils, except in the 45-y sites. The relative abundance of Actinobacteria and Proteobacteria in the rhizosphere soils increased first and then decreased, peaking at the 22-y sites and 45-y sites, respectively. Chloroflexi and Firmicutes significantly (*p <* 0.05) increased with increasing stand age (Figure 3A; Table S2). However, the relative abundance of Actinobacteria, Proteobacteria, and Firmicutes in the bulk soils significantly (*p <* 0.05) increased along the teak plantation chronosequence, peaking at 45-y and 55-y sites. Acidobacteria and Chloroflexi in the bulk soils decreased with increasing stand age (Figure 3B; Table S2).

**Figure 4:**
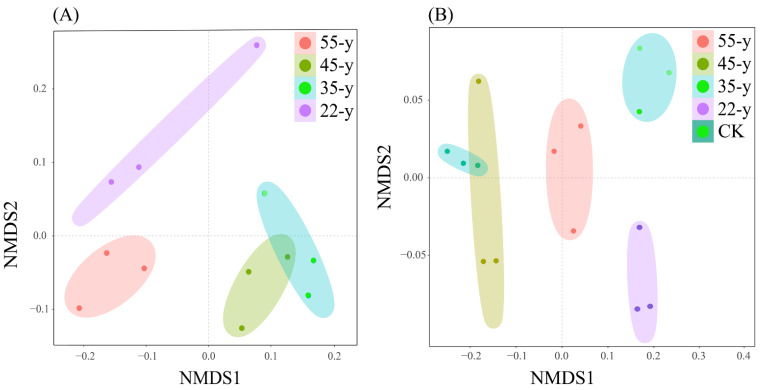
Non-metric multidimensional scaling (NMDS) ordination showing the structure differences of bacterial communities in rhizosphere soil (**B**) and soil (**A**) communities among the teak plantations.

The NMDS ordination showed that soil samples in the rhizosphere and bulk soils were differentiated into four and three clusters, respectively. The sample sites from the 45-y sites and 35-y sites were similar to each other (Figure 4). In addition, ANOSIM and PERMANOVA confirmed that bacterial community structures differed significantly among different teak plantations in the rhizosphere (ANOSIM: R = 0.934, *p* = 0.001; PERMANOVA: R^2^ = 0.740, *p* = 0.001) and bulk (ANOSIM: R = 0.633, *p* = 0.002; PERMANOVA: R^2^ = 0.512, *p* = 0.001) soils, which supported the results of NMDS ordination analysis (Table S3).

### 3.4. Soil Bacterial Co-Occurrence Networks

**Figure 5:**
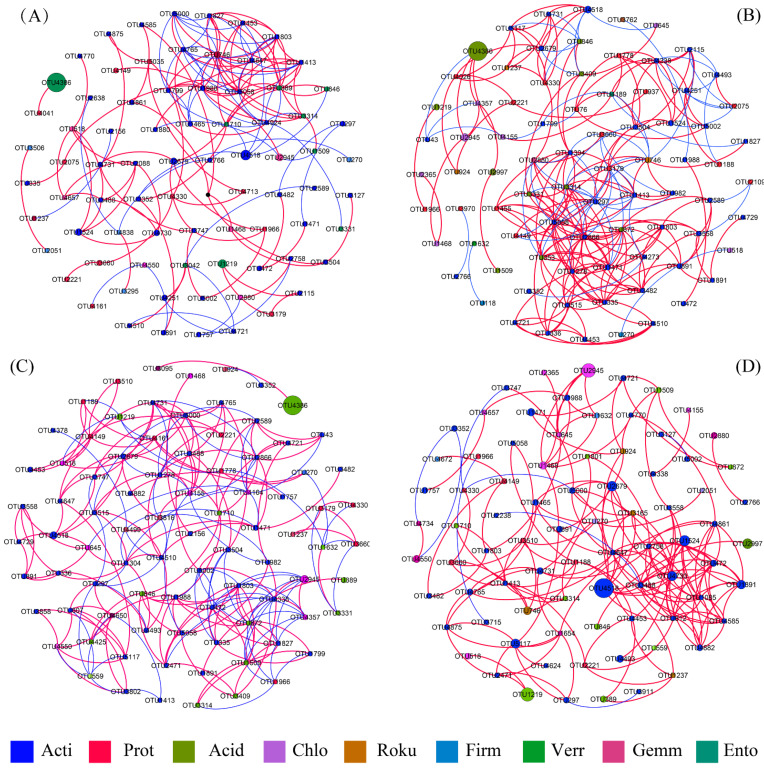
Soil bacterial co-occurrence network of teak with different growth stages ((**A**), 22-y; (**B**), 35-y; (**C**), 45-y; (**D**), 55-y). The nodes were colored based on phylum levels. A connection stands for a strong (Pearson’s correlation coefficient R > 0.6) and significant (*p <* 0.01) correlation. The size of each node is proportional to the abundance of OTUs, the thickness of each edge is proportional to the value of Pearson’s correlation coefficient. Acti: Actinobacterial; Prot: Proteobacteria; Acid: Acidobacteria; Chlo: Chloroflexi; Roku: Rokubacteria; Firm: Firmicutes; Verr: Verrucomicrobia; Gemm: Gemmatimonadetes; Ento: Entotheonellaeota.

Four networks were generated by the construction of correlation-based networks of the bacterial communities, including 79, 91, 86, and 81 nodes connected by 144, 237, 221, and 172 edges, respectively (Figure 5; Table 3). The number of structural features (nodes and edges) differed strikingly within each network, as shown by an initial increase and then decline, peaking at 35-y sites. The average clustering coefficient (ACC) at 35-y sites was also higher than at other sites. In contrast, the average path length (APL) at 55-y sites was the highest among the different stand ages. The hubs (highly connected nodes, i.e., degree) in the 35-y sites were OTU 2866, OTU 5058, OTU 1278, OTU 297, OTU 1471, and OTU 872, which belonged to Actinobacteria and Proteobacteria. Actinobacteria and Chloroflexi in 22-y sites, Actinobacteria and Proteobacteria in 45-y sites, and Actinobacteria in 55-y sites were the major taxa for the respective sites. 

### 3.5. Relationships between Soil Properties and Soil Bacterial Community

**Figure 6:**
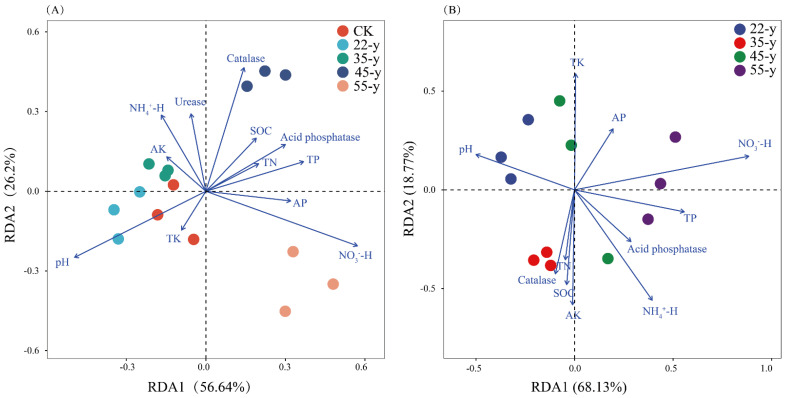
The effects of soil factors on soil bulk (**B**) and rhizosphere (**A**) bacterial community structure identified by RDA. TN: total nitrogen; TP: total phosphorous; TK: total potassium; AP: available phosphorous; AK: available potassium; ${\mathrm{NO}}_{3}^{-}$-H: nitrate nitrogen; ${\mathrm{NH}}_{4}^{+}$-H: ammonium nitrogen; SOC: soil organic carbon.

**Figure 7:**
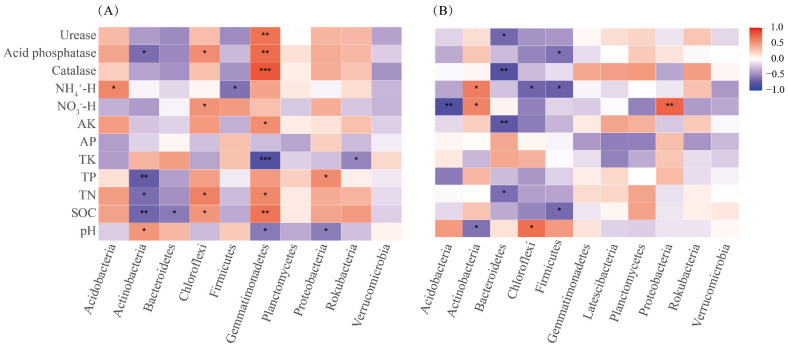
Correlation heat map between soil chemical properties and bacterial community (phyla level) of teak plantations with different stand ages in rhizosphere (**A**) and bulk (**B**) soils. * *p <* 0.05; ** *p <* 0.01; *** *p <* 0.001.

${\mathrm{NO}}_{3}^{-}$-H, pH, and catalase were the main factors influencing the bacterial community structure in rhizosphere soils (Figure 6A). ${\mathrm{NO}}_{3}^{-}$-H, pH, SOC, TK, TP, AP, AK, and urease significantly (*p* < 0.05) contributed to the bacterial community structure in the rhizosphere soils (Table 4). The pH, SOC, TK, AP, and TK significantly affected the bacterial community structure in the bulk soils (Table 4). The correlation heat map showed that the abundance of bacterial phyla in the rhizosphere soil was not significantly related to soil AP (*p* > 0.05). While the ${\mathrm{NO}}_{3}^{-}$-H, pH, and ${\mathrm{NH}}_{4}^{+}$-H significantly affected the bacterial community structure in the bulk soil (Figure 7). Spearman’s correlation test showed that the Simpson’s index of rhizosphere soil bacterial community was significantly (*p <* 0.05) positive for AK, catalase, and urease, but significantly negative for pH (Table S4). The alpha diversity of the bacterial community in the bulk soil was related to TP, AK, ${\mathrm{NH}}_{4}^{+}$-H, catalase, and urease (Table S4).

## 4. Discussion

### 4.1. Responses of Soil Properties to the Chronosequence

Afforestation is an effective restoration technique that can be used to increase vegetation and biodiversity for the restoration of degraded forest ecosystems [43]. Afforestation and vegetation succession can reinforce soil organic carbon and nitrogen cycles by increasing soil organic matter input and decreasing the decomposition rate [8]. In the present study, pH, SOC, TN, ${\mathrm{NO}}_{3}^{-}$-H, TP, and TK significantly (*p <* 0.05) changed along gradients of forest age (Table S1), which suggests that the cycling and turnover of these nutrients were stimulated with the increase in stand age. Furthermore, soil properties differ greatly between the rhizosphere and bulk soil in teak plantations. For instance, the urease, acid phosphatase, ${\mathrm{NH}}_{4}^{+}$-H, AK, and AP in the rhizosphere soil were higher than those in the bulk soil (Table S1). Li et al. [44] reported similar findings regarding the effect of seasonal variation on microbial community structures in *Camellia yuhsienensis*. It is reasonable to accept that soil biological (microbial and enzymatic) activities are highly related to soil physicochemical properties [15]. Thus, higher urease and acid phosphatase activities in the rhizosphere soil may result in higher nutrient content due to participation in the conversion of soil nutrients.

### 4.2. Shifts in Bacterial Communities between Successional Series

Soil microbial diversity is an essential indicator that reflects soil functionality and health [45,46]. Huang et al. [47] revealed that higher microbial diversity could be conducive to maintaining stable agricultural ecosystems and sustainable crop production. Studies have reported that soil fungal and bacterial diversity significantly increased along a gradient of stand age in *Pinus sylvestris* [48] and *Robinia pseudoaccacia* [49]. This phenomenon may be attributed to the accumulation of soil nutrients derived from the decomposition of the litter and root exudates as vegetation biomass gradually increases [48]. The accumulated matter could provide more substrates for the decomposition and growth of different bacterial species. However, the bacterial community diversity in this study showed no statistical difference among stand ages in either the rhizosphere soil samples or bulk soil samples (Table 2). This is consistent with the results reported by Liu et al. [19], who found that the microbial alpha diversity showed no significant differences along a *Pinus tabulaeformis* plantation chronosequence. Ezeokoli et al. [50] also reported that the diversity of arbuscular mycorrhizal fungal communities in the soil and roots were not significantly different along a post-coal mining reclamation chronosequence in South Africa. This phenomenon may be ascribed to a large variation in the obtained sequence abundance between the samples. Thus, it is necessary to deal with the inequalities by standardizing samples via rarefaction to a common sequencing depth per sample [51]. The diversity and richness of the data obtained from high-throughput sequencing may, therefore, not represent the true soil bacterial community [52]. The diversity (Shannon and Simpson) and richness (Sobs and Chao 1) indices in the bulk soil were significantly higher than those in the rhizosphere soils (Figure 1), which was consistent with the results reported by Wang et al. [53]. This phenomenon may be explained by the host plants exerting a selective force on the rhizosphere microbiome, resulting in the differentiation of bacterial communities between bulk soil and rhizosphere soils [54]. In addition, root activities directly affect carbon availability, regulate pH, and increase urease and acid phosphatase activity and nutrient content in the rhizosphere soils compared to those in the bulk soils (Table S1). These differences may reduce heterogeneity in the rhizosphere soil environment, resulting in a decline in bacterial community alpha diversity in rhizosphere soils [53].

The NMDS ordination and similarity analysis (ANOSIM and PERMANOVA) showed that the composition and structure of the bacterial communities were statistically different along a teak plantation chronosequence (Figure 4; Table S3). Numerous studies have reported that the structure of the bacterial community is influenced by the planting years of woody plants [5,19]. Among the teak plantations, the dominant phyla in the bacterial communities were Actinobacteria, Proteobacteria, and Acidobacteria, regardless of whether rhizosphere or bulk soil samples were scrutinized (Figure 2, Table S2), which was similar to the results of previous studies [55]. Earlier studies reported that soil microbes can change the composition and structure of corresponding microbial communities to adapt to the shift in environmental conditions during succession [10,56]. Our results also indicate that the divergent responses of bacterial taxa progressed with stand age (Table S2), such as the relative abundance of Actinobacteria in the rhizosphere soils that first increased and then declined with stand age. Studies have revealed that Actinobacteria prefer nutrient-poor environments and are considered oligotrophic species [19,21]. These bacteria are often dominant in dry, low nutrient, and barren conditions [57,58]. The increase in Actinobacteria abundance may, therefore, be related to the decline in rhizosphere soil nutrients. The relative abundance of Actinobacteria in the bulk soils increased with chronosequence, which may correlate with the shift in pH in the bulk soils. A previous study reported that Actinobacteria also thrive under acidic conditions [49]. The heat map shows that the abundance of Actinobacteria is significantly (*p <* 0.05) negatively correlated with pH (Figure 7B), which supports our hypothesis that soil pH may be a vital environmental factor affecting the soil bacterial community. In contrast, Proteobacteria belong to the symbiotic or r-strategy taxa and have a remarkable competitive advantage under nutrient-rich and high-carbon conditions [5,49]. Previous studies reported that the abundance of Proteobacteria increased due to the accumulation of nutrients with succession [59,60]. We observed a similar phenomenon in this study. 

We further observed a clear conversion in Acidobacteria dominance, with these bacteria dominating in the young-growth stage in the bulk soils, while they were dominant in the old-growth stage in the rhizosphere soils (Figure S1). The underground vegetation communities among different stand ages of the teak plantation were not the same, and the conversion may be affected by different vegetation types. Deng et al. [21] also reported that different revegetation forest stands possessed dissimilar bacterial communities, and the relative abundances of dominant bacterial phyla were affected by different revegetation types.

### 4.3. Correlation between Bacterial Community and Edaphic Factors

The relationship between bacterial communities and edaphic factors in teak plantations of different stand ages was analyzed using RDA and Mantel tests (Figure 6; Table 4). The results showed that pH had a significant impact on the bacterial community structure in the rhizosphere and bulk soil. Substantial studies have revealed that pH is a key regulator in shaping the distribution of soil bacterial communities [10,61,62,63]. The strong linkage between soil pH and microbial communities could be attributed to the narrow pH range for the optimal growth of bacteria [8,63]. Hence, a slight change in pH might cause microbes to respond quickly. Moreover, the effects of pH on the structures of the microbial communities may be associated with their ability to mediate nutrient availability [62]. pH has a major impact on the mobility of multiple compounds in the soil and affects many connected biological processes [64]. SOC has been considered as the major factor influencing microbial community structure, especially bacteria [63,65]. The present study also shows that SOC has a significant impact on the bacterial community structure in bulk soils. Our results show that AK and TK have a significant impact on bacterial community structure, especially in bulk soils, which is consistent with the results reported by Ma et al. [24], who reported a statistical correlation between microbial structure and soil TK in *Eucalyptus urophylla* plantations. Leaching of K from the soil may explain this phenomenon [9]. Our experiment was conducted in a tropical forest region, and heavy rainfall may emphasize this phenomenon. K is necessary for the majority of plants and microbes to maintain vital metabolic processes, such as ATP production, photosynthesis, and protein synthesis [66]. Therefore, the lack of K not only directly affects the microbial communities but also indirectly limits the absorption and utilization of other nutrients by plants. In this sense, teak trees may facilitate the shift of bacterial communities towards a community with an increased ability to mobilize soil K [24]. Moreover, AP and TP were significantly correlated with bacterial community structure in this study. Most microorganisms need P for the synthesis of ribosomal RNA based on the growth rate hypothesis and this could explain the close relationship between P and bacterial community structure [5,36]. Urease is involved in soil N cycling Many studies have reported that N is a crucial factor affecting soil bacterial communities [7,59]. We also observed that urease and ${\mathrm{NO}}_{3}^{-}$-H contributed to the shift in the bacterial community structure along a chronosequence. Catalase can decompose H_2_O_2_ and protect host plants and microbes from oxidative damage [67]. The bacterial community structure in the bulk soils was influenced by catalase, which suggested that the bacterial community structure was related to the ability of catalase to induce oxidative stress.

Taken together, these results indicate that the shift in edaphic factors along a chronosequence affects the soil bacterial community structure. Edaphic factors and vegetation characteristics, such as community, diversity, and productivity are the two main drivers of soil microbial community structure [7]. A prior study reported that bacterial communities could be driven by changes in vegetation during secondary succession [68]. Liu et al. [69] also found that plant species richness was significantly correlated with a shift in the bacterial community structure. The underlying mechanism could be attributed to changes in plant community composition, and this shift may alter root exudates and litter [6]. The litter and root exudates may provide more substrates for bacterial growth and development. Furthermore, microbial communities can be indirectly affected by vegetation communities through the alteration of the soil properties. Some researchers have pointed out that rhizosphere microbial communities are mostly influenced by plant characteristics, whereas those in bulk soil are strongly related to the soil properties. Since the rhizosphere microbiome is closer to the roots, it can be directly influenced by the root exudation of the plants, whereas the microbiome in bulk soils obtains nutrients mainly from the substrate [36]. Based on these results, it is necessary to investigate the plant community characteristics in future studies to better understand the drivers of bacterial divergence between rhizosphere and bulk soil bacterial community structure over chronosequence.

### 4.4. Co-Occurrence Network of the Bacterial Communities

Correlation-based network analysis can provide new insights into the rules of microbial community assembly, reflecting ecological processes and interactions between microbes [70,71,72]. We found that the bacterial network at 35-y sites was more complex and stable than at other sites in terms of topological properties, such as nodes, edges, and average degree (Table 3). Consistent with our results, a previous study also revealed that the nodes and edges of the network differed drastically during secondary succession, exhibiting a trend of increasing first and then decreasing [73]. This finding, coupled with our results, could indicate that the relationship among the soil bacteria could change with succession to adapt to the shift in the environment [74]. We hypothesize that the greater number of interactions among bacterial species at the 35-y sites helped them perform better functions, such as those involved in nutrient cycling, promoting plant growth [33,74], and maintaining plant health, as the complexity of the microbiome has been speculated to predict ecosystem functions [75,76]. Table 2 shows that the content of most soil parameters reached their maximum value in the 35-y sites, including SOC, TN, TP, AK, ${\mathrm{NH}}_{4}^{+}$-H, and catalase, which partially supports our hypothesis that these interactions among bacteria may be involved in soil nutrient cycling. Several studies have explained modules as niches [70,77], and higher modularity values indicate strong niche differentiation. Thus, the presence of more niches for the soil bacterial community in the 45-y sites may provide more opportunities for different bacterial species to interact with each other [74].

The number of strongly positive correlations (65.28%, 73.84%, 64.71%, and 95.35% in the 22-y sites, 35-y sites, 45-y sites, and 55-y sites, respectively) per network was much higher than negative correlations (Figure 5). This indicates that most soil bacterial taxa may synergistically act or share similar ecological niches in the soil environment of teak plantations [72]. In addition, an increase in positive associations was observed with stand age, suggesting that the co-occurrence network between synergistic bacterial groups was enhanced [72,78]. We also found that the average path length (APL) value in the 45-y sites was the smallest, indicating that it was a small-world network [74]. The small-world network indicates that the bacterial community can respond quickly to changes in external environmental conditions [78]. Network analysis can not only be applied to determine how species occur together in niches but can also identify the keystone taxa that play a critical role in communities [79,80]. It has been proposed that nodes with high betweenness centrality (BC) scores are crucial for maintaining network stability and structure [20,29,81]. Based on the BC scores, OTU 4647, OTU 2679, and OTU 4731 at the 22-y sites; OTU 746, OTU 4189, and OTU 4155 at the 35-y sites; OTU 297, OTU 1710, and OTU 1278 at the 45-y sites; OTU 5000, OTU 4330, and OTU 3660 at the 55-y sites, were identified as the top-three keystone taxa at each site. Therefore, we guessed that they might play a significant role in maintaining the structure and function of soil bacterial communities in teak plantations. Additionally, the keystone taxa were different in each network over the chronosequence observed in the present study. This may mean the realignment of species interactions within the bacterial community [73].

### 4.5. Implications of Bacterial Community Variation in Teak Plantations

Underground ecological processes, nutrient turnover rates, and metabolic rates in tropical forests are much stronger than those in other ecosystems [48]. These processes include the decomposition rate of humus, litter, and root exudates mediated by soil microbes. To our knowledge, this is the first study to characterize the shift in soil bacterial communities and co-occurrence patterns along a teak plantation chronosequence using sequencing technology. Our study revealed that the diversity of the bacterial community was not significantly (*p* > 0.05) different among the different stand age groups. We were not able to show that the diversity of the bacterial community shifts along a chronosequence. The maximum or minimum bacterial community diversity may have occurred in the young stage (<22 years old) of teak plantations. However, our research showed that the topological properties of the network, such as nodes, edges, and average degree, first increased and then declined, indicating that the complexity and stability of the network decreased in the later stages of succession. The diversity and complexity of microbial communities are closely correlated with ecosystem functions and services [55]. Therefore, maintaining a higher diversity and complexity of the bacterial communities may be critical for ecosystem productivity.

Microbes present in the rhizosphere can have a profound effect on the growth, nutrition, and health of plants in ecosystems [82]. Actinobacteria are gram-positive bacteria that play a vital role in the decomposition of organic matter and the soil carbon cycle, such as cellulose and chitin [83]. Actinobacteria can also degrade dead woody-mass to provide carbon to the associated mycorrhizae as mycorrhizae helper bacteria [73]. The abundance of Actinobacteria in the rhizosphere soils increased first and then decreased significantly, which suggested that the microbial decomposition process for dead woody-mass, cellulose, and chitin changes with stand age. Proteobacteria taxa are mainly involved in fixing nitrogen from the atmosphere [7]. Acidobacteria can participate in iron cycling in soils [84] and degrade residue polymers in plants [85]. Chloroflexi can acquire energy and fix carbon dioxide through photosynthesis [86]. The shift of these bacterial taxa with stand age also indicated variations in the corresponding functions. Furthermore, litter gradually accumulated with forest age. Our results showed that the abundance of Firmicutes in the rhizosphere soils increased with stand age, suggesting that Firmicutes may play a crucial role in litter decomposition [48]. Overall, our research revealed that the composition and functions of soil bacterial communities changed with stand age. Although we have confirmed that variation in soil bacterial community structures and co-occurrence patterns changed with chronosequence, it is difficult to replicate this study in other ecosystems, as the sites of each type of forest succession were less than 100 m. However, our results are essential for the cultivation of and could provide new insights into the management of teak plantations.

## 5. Conclusions

Our results showed that the stand age had no significant (*p* > 0.05) impact on the alpha diversity of bacterial communities in rhizosphere and bulk soils. However, there are significant (*p <* 0.05) differences in soil bacterial community structure in teak plantations. Topological features of the co-occurrence network showed that the number of nodes and edges first increased and then decreased with stand age. In the rhizosphere soils, the soil bacterial community was mainly regulated by urease, TK, and pH, whereas the bacterial community in the bulk soils was mainly affected by TK, AK, AP, pH, and catalase. Overall, our results indicate that soil nutrients play a leading role in shaping the soil bacterial community structure. The evaluation of bacterial community structure and composition is only a first step in teak plantations. Future work should focus on obtaining highly efficient functional and functionally complementary microbe strains and applying them to teak plantations may be the key to improving the productivity of teak plantations.

## Figures and Tables

**Table 1:** Information about experiment sites and teak plantations.

Stand Age	Longitude (N)	Latitude (E)	Altitude (m)	Mean DBH (m)	Mean Height (m)	Canopy Closure (%)
CK	18°42′16.14″	108°47′46.78″	118	-	-	-
22-y	18°42′20.93″	108°48′57.56″	161	0.20	15.44	0.65
35-y	18°41′52.93″	108°47′2.09″	106	0.33	23.66	0.70
45-y	18°41′58.85″	108°47′5.47″	83	0.31	20.58	0.67
55-y	18°42′11.48″	108°49′28.27″	154	0.38	24.00	0.75

Values are mean (*n* = 3). 22-y: 22 year-old stand; 35-y: 35 year-old stand; 45-y: 45 year-old stand; 55-y: 55 year-old stand; CK: control (the adjacent native grassland without teak plantation). DBH: diameter at 1.3 m breast height.

**Table 2:** Differences in bacterial diversity between rhizosphere and bulk soil along a teak plantation chronosequence.

Alpha Diversity	Soil	CK	22-y	35-y	45-y	55-y	Age
Sobs	R	2057.00 ± 60.48	2037.00 ± 32.47	2087.00 ± 56.36	2063.00 ± 34.70	2241.33 ± 68.13	ns
	B	-	2378.67 ± 19.03	2369.67 ± 95.57	2293.33 ± 50.52	2185.67 ± 168.03	ns
Shannon	R	5.85 ± 0.05	6.02 ± 0.01	5.99 ± 0.06	5.96 ± 0.04	6.00 ± 0.09	ns
	B	-	6.18 ± 0.04	5.93 ± 0.10	5.94 ± 0.10	5.87 ± 0.16	ns
Simpson	R	0.02 ± 0.00	0.01 ± 0.00	0.01 ± 0.00	0.02 ± 0.00	0.02 ± 0.00	ns
	B	-	0.01 ± 0.00	0.02 ± 0.00	0.02 ± 0.00	0.02 ± 0.00	ns
Chao 1	R	2562.04 ± 84.45	2561.24 ± 54.80	2582.40 ± 53.85	2595.71 ± 67.34	2791.59 ± 38.01	ns
	B	-	3070.74 ± 49.04	3081.95 ± 94.98	3073.89 ± 25.80	2967.665 ± 172.92	ns
Coverage (%)	R	98.25	97.98	98.36	98.13	98.06	
	B		97.84	97.94	97.68	97.70	

Values are presented as the mean (*n* = 3) ± stand error except the coverage is presented as the mean. ns indicate non-significant differences among different stand ages at *p <* 0.05 level.

**Table 3:** Topological characteristics of co-occurrence network of soil bacterial communities of teak plantations with different growth stages.

Treatments	Nodes	Edges	Average Path Length (APL)	Modularity (MD)	Average Clustering Coefficient (ACC)	Average Degree
22-y	79	144	3.981	1.683	0.554	3.646
35-y	91	237	4.134	0.965	0.591	5.852
45-y	86	221	3.511	1.828	0.581	5.140
55-y	81	172	5.960	0.674	0.574	4.247

**Table 4:** Relationship between the relative abundance of OTUs and variables as tested by Mantel analysis.

Variables	Bulk		Rhizosphere	
	R^2^	*p*	R^2^	*p*
pH	0.327	0.019 *	0.264	0.007 **
SOC	0.287	0.032 *	−0.040	0.611
TN	0.058	0.292	0.029	0.347
TP	−0.116	0.756	0.213	0.024 *
TK	0.351	0.009 **	0.335	0.001 **
AP	0.343	0.012 *	0.174	0.038 *
AK	0.472	0.001 **	0.195	0.035 *
${\mathrm{NO}}_{3}^{-}$-H	0.116	0.229	0.180	0.045 *
${\mathrm{NH}}_{4}^{+}$-H	0.090	0.260	−0.029	0.570
Catalase	0.344	0.011 *	0.015	0.361
Acid phosphatase	0.225	0.079	0.023	0.354
Urease	0.011	0.434	0.585	0.001 **

SOC: soil organic carbon; TN: total nitrogen; TP: total phosphorous; TK: total potassium; AP: available phosphorous; AK: available potassium; ${\mathrm{NO}}_{3}^{-}$-H: nitrate nitrogen; ${\mathrm{NH}}_{4}^{+}$-H: ammonium nitrogen. * *p <* 0.05; ** *p <* 0.01.

## Data Availability

The data presented in this study are available are available in the NCBI Sequence Read Achieve database (accession number: PRJNA752823).

## Supplementary Materials

The following are available online at https://www.mdpi.com/article/10.3390/biology10121329/s1, Table S1: Chemical, enzyme activities characteristics of the rhizosphere and bulk soil., Table S2: Relative abundances of bacterial dominant phyla in both rhizosphere and bulk soils along a chronosequence of stand age gradients., Table S3: Analysis of similarities (ANOSIM) and PERMANOVA (permutation multivariate analysis of variance) based on the Bray-Curtis distance showing the differences in bacterial community compositions in the rhizosphere and bulk soils along a chronosequence. Table S4: Spearman’s correlation between bacterial alpha diversity and soil properties, Figure S1: Differences in soil bacterial dominant phyla between the rhizosphere and bulk soils of the teak plantations.

## Abbreviations

22-y	22-year-old stand
35-y	35-year-old stand
45-y	45-year-old stand
55-y	55-year-old stand
CK	control
DBH	diameter at breast height
OTUs	Operational taxonomic units
RDP	Ribosomal Database Project
SOC	Soil organic carbon
TN	Total nitrogen
TP	Total phosphorus
TK	Total potassium
AP	Available phosphorus
AK	Available potassium
${\mathrm{NH}}_{4}^{+}$-H	Ammonium-N
${\mathrm{NO}}_{3}^{-}$-H	Nitrate-N
HSD	Honestly significant difference
NMDS	Non-metric multidimensional scaling
ANOSIM	Analysis of similarities
PERMANOVA	Permutation multivariate analysis of variance
RDA	Redundancy analysis
APL	Average path length
MD	Modularity
ACC	Average clustering coefficient
BC	Betweenness centrality
