# Supplementary material for: Soil Bacterial Community Shifts Are Driven by Soil Nutrient Availability along a Teak Plantation Chronosequence in Tropical Forests in China
Source: Biology (Basel). 2021 Dec 15;10(12):1329. doi: 10.3390/biology10121329 (PMC8698287; doi:10.3390/biology10121329)
Supplement: Supplementary file 1 [file biology-10-01329-s001.zip › biology-1461706-supplementary.pdf]

biology

MDPI

Article

Soil Bacterial Community Shifts Are Driven by Soil Nutrient Availability along a Teak Plantation Chronosequence in Tropical Forests in China

Zhi Yu, Kunnan Liang, Guihua Huang, Xianbang Wang, Mingping Lin, Yinglong Chen and Zaizhi Zhou

Table S1. Chemical, enzyme activities characteristics of the rhizosphere and bulk soil.

Soil properties	Soil	CK	22-y	35-y	45-y	55-y
pH	R	6.56±0.02b	6.77±0.01a	6.52±0.06b	6.54±0.02b	6.56±0.02b
	B	-	6.61±0.03a	6.38±0.03a	6.51±0.03a	6.18±0.53a
SOC (g/kg)	R	29.16±3.14b	26.07±1.95b	46.98±6.86a	34.71±1.94b	35.42±1.96b
	B	-	22.50±3.88b	36.02±7.04a	23.17±2.22b	26.26±3.95ab
TN (g/kg)	R	1.71±0.091bc	1.45±0.07c	2.70±0.33a	1.93±0.18bc	2.09±0.17b
	B	-	1.38±0.31a	2.22±0.58a	1.71±0.08a	1.59±0.30a
TP (g/kg)	R	0.28±0.01ab	0.25±0.04b	0.36±0.07a	0.32±0.01ab	0.33±0.03ab
	B	-	0.22±0.01a	0.32±0.05a	0.31±0.01a	0.36±0.13a
TK (g/kg)	R	32.24±1.53ab	38.60±0.30a	21.00±0.58b	31.89±4.51ab	31.45±9.35ab
	B	-	36.71±1.76a	21.69±0.82b	31.05±0.23ab	31.71±9.27ab
AP (mg/kg)	R	2.30±0.10a	3.31±0.87a	1.99±0.57a	3.56±0.86a	3.44±1.40a
	B	-	2.80±0.40a	1.48±0.48a	2.17±0.32a	2.87±1.47a
AK (mg/kg)	R	151.32±16.95b	78.80±13.57c	308.64±34.54a	129.18±30.41bc	134.22±17.84bc
	B	-	64.53±9.19c	231.28±13.03a	131.89±24.47b	117.92±37.71bc
NO_3^--H (mg/kg)	R	15.71±2.09a	13.98±2.74a	15.19±0.59a	16.57±2.59a	19.16±3.73a
	B	-	10.53±0.29c	13.12±3.04bc	17.09±1.04ab	20.71±2.26a
NH_4^+-H (mg/kg)	R	11.22±0.79a	9.49±6.18a	22.44±2.34a	13.98±6.30a	10.87±6.46a
	B	-	3.97±0.30a	16.57±4.75a	8.46±4.67a	13.12±7.40a
Catalase	R	14.19±1.10bc	8.43±0.47c	20.67±2.95a	17.54±3.12ab	13.17±2.87bc
(ml/g)	B	-	7.43±2.31b	22.45±2.15a	19.49±0.95a	9.93±3.61b
Acid phosphatase	R	0.53±0.02c	0.43±0.04d	0.99±0.12a	0.70±0.08bc	0.74±0.09b
(mg/g)	B	-	0.35±0.08a	0.61±0.14a	0.39±0.07a	0.54±0.14a
Urease	R	1.56±0.07b	1.13±0.27b	8.46±2.38a	3.76±1.39b	2.61±1.50b
(mg/g)	B	-	0.77±0.12a	2.98±2.59a	1.31±0.57a	1.49±1.03a

Values are represented as the mean (n=3) ± the standard deviation. Different lowercase letters indicate statistical differences ($P < 0.05$) among different stand ages based on a one-way ANOVA followed by a Tukey's HSD test. TOC: soil total organic carbon; TN: total nitrogen; TP: total phosphorous; TK: total potassium; AP: available phosphorous; AK: available potassium; NO_3^--H: nitrate nitrogen; NH_4^+-H: ammonium nitrogen. R: rhizosphere soil; B: bulk soil.

Table S2. Relative abundances of bacterial dominant phyla in both rhizosphere and bulk soils along a chronosequence of stand age gradients.

Phyla	Soil	CK	22-y	35-y	45-y	55-y
Acti	R	40.11	47.60	38.70	26.68	24.57
	B	-	21.61	29.381	26.16	32.08
Prot	R	25.13	21.35	24.49	42.00	30.99
	B	-	32.19	30.58	34.11	43.56
Acid	R	12.50	11.63	15.25	11.03	12.71
	B	-	18.97	16.57	9.47	4.56
Chlo	R	7.16	5.48	7.65	5.80	9.55
	B	-	9.39	5.447	7.35	5.59
Roku	R	5.21	4.81	5.87	6.42	4.71
	B	-	5.61	7.12	6.75	5.04
Firm	R	3.12	2.11	1.30	1.96	10.33
	B	-	4.29	2.82	8.73	3.03
Verr	R	1.42	1.85	1.27	0.81	1.66
	B	-	2.27	2.04	1.41	0.86
Bact	R	1.76	1.54	1.12	1.04	1.40
	B	-	1.41	0.76	0.76	1.73
Gemm	R	0.98	0.83	1.42	1.50	1.03
	B	-	1.15	1.21	2.08	1.09
Plan	R	1.01	0.92	1.14	0.89	1.08
	B	-	1.47	2.07	1.10	0.58

Values are mean (n=3). R: rhizosphere; B: bulk. Acti: Actinobacteria, Prot: Proteobacteria, Acid: Acidobacteria, Chlo: Chloroflexi, Roku: Rokubacteria, Firm: Firmicutes, Verr: Verrucomicrobia, Bact: Bacteroidetes, Gemm: Gemmatimonadetes, Plan: Planctomycetes.

Table S3. Analysis of similarities (ANOSIM) and PERMANOVA (permutation multivariate analysis of variance) based on the Bray-Curtis distance showing the differences in bacterial community compositions in the rhizosphere and bulk soils along a chronosequence.

Type	df	PERMANOVA		ANOSIM	
		R^2	*P*	*R*	*P*
Rhizosphere soil	4	0.740	**0.001**	0.935	**0.001**
Bulk soil	3	0.633	**0.002**	0.633	**0.002**

Table S4. Spearman's correlation between bacterial alpha diversity and soil properties.

Soil properties	Rhizosphere				Bulk			
	Sobs	Shannon	Simpson	Chao	Sobs	Shannon	Simpson	Chao
pH	0.15	0.47	**-0.54***	0.19	0.22	0.47	-0.55	-0.21
SOC	0.16	0.05	0.34	0.04	-0.31	-0.41	0.46	-0.15
TN	0.17	-0.06	0.44	0.07	-0.35	-0.50	0.56	-0.30
TP	0.22	-0.06	0.42	0.26	**-0.64***	-0.50	**0.67***	-0.10
TK	-0.13	-0.05	-0.18	-0.07	0.25	0.21	-0.57	-0.41
AP	0.23	0.21	-0.07	0.36	0.00	-0.01	-0.39	-0.41
AK	0.04	-0.43	**0.63***	-0.08	-0.21	**-0.65***	**0.63***	-0.01
NO_3^--H	0.46	0.11	0.38	**0.69****	-0.47	-0.56	0.38	-0.10
NH_4^+-H	-0.05	-0.19	0.41	-0.10	-0.26	**-0.77****	**0.71***	-0.13
Catalase	0.07	-0.21	**0.57***	0.04	-0.18	-0.48	**0.62***	-0.03
Acid phosphatase	0.30	0.09	0.36	0.22	-0.37	-0.48	0.48	-0.16
Urease	0.04	-0.23	**0.55***	-0.04	-0.23	**-0.76****	0.50	-0.36

SOC: soil organic carbon; TN: total nitrogen; TP: total phosphorous; TK: total potassium; AP: available phosphorous; AK: available potassium; NO_3^--H: nitrate nitrogen; NH_4^+-H: ammonium nitrogen. * $P < 0.05$; ** $P < 0.01$.

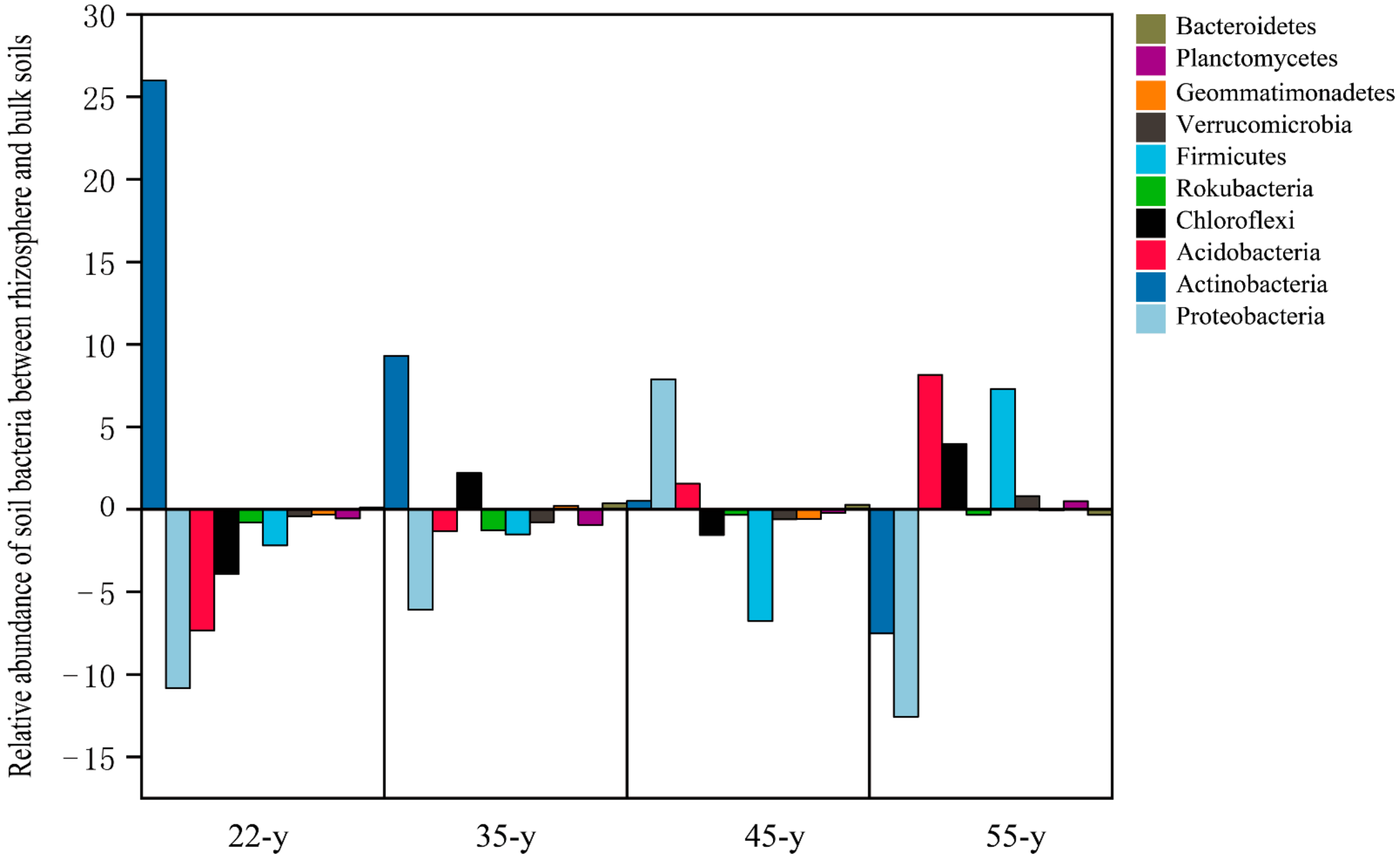

Figure S1. Differences in soil bacterial dominant phyla between the rhizosphere and bulk soil of the teak plantations.
